# High level of HIV-1 drug resistance mutations in patients with unsuppressed viral loads in rural northern South Africa

**DOI:** 10.1186/s12981-017-0161-z

**Published:** 2017-07-27

**Authors:** Elizabeth M. Etta, Lufuno Mavhandu, Cecile Manhaeve, Keanan McGonigle, Patrick Jackson, David Rekosh, Marie-Louise Hammarskjold, Pascal O. Bessong, Denis M. Tebit

**Affiliations:** 1 HIV/AIDS & Global Health Research Program, Department of Microbiology, University of Venda, Thohoyandou, 0950 South Africa; 2 Bela-Bela HIV/AIDS Prevention Group Wellness Clinic (HAPG), Bela-Bela, South Africa; 3 Department of Microbiology, Immunology and Cancer Biology, Myles H. Thaler Center for AIDS and Human Retrovirus Research, University of Virginia, Charlottesville, VA 22908 USA

**Keywords:** HIV drug resistance, Antiretroviral therapy, Peripheral blood mononuclear cells, Plasma, Rural South Africa

## Abstract

**Background:**

Combination antiretroviral therapy (cART) has significantly reduced HIV morbidity and mortality in both developed and developing countries. However, the sustainability of cART may be compromised by the emergence of viral drug resistance mutations (DRM) and the cellular persistence of proviruses carrying these DRM. This is potentially a more serious problem in resource limited settings.

**Methods:**

DRM were evaluated in individuals with unsuppressed viral loads after first or multiple lines of cART at two sites in rural Limpopo, South Africa. Seventy-two patients with viral loads of >1000 copies/ml were recruited between March 2014 and December 2015. Complete protease (PR) and partial Reverse Transcriptase (RT) sequences were amplified from both plasma RNA and paired proviral DNA from 35 of these subjects. Amplicons were directly sequenced to determine subtype and DRM using the Stanford HIV Drug Resistance Interpretation algorithm.

**Results:**

Among the 72 samples, 69 could be PCR amplified from RNA and 35 from both RNA and DNA. Sixty-five (94.2%) viruses were subtype C, while one was subtype B (1.4%), one recombinant K/C, one recombinant C/B and one unclassified. Fifty-eight (84%) sequences carried at least one DRM, while 11 (15.9%) displayed no DRM. DRM prevalence according to drug class was: NRTI 60.8% NNRTI 65.2%, and PI 5.8%. The most common DRMs were; M184V (51.7%), K103N (50%), V106M (20.6%), D67N (13.3%), K65R (12%). The frequency of the DRM tracked well with the frequency of use of medications to which the mutations were predicted to confer resistance. Interestingly, a significant number of subjects showed predicted resistance to the newer NNRTIs, etravirine (33%) and rilpivirine (42%), both of which are not yet available in this setting. The proportion of DRM in RNA and DNA were mostly similar with the exception of the thymidine analogue mutations (TAMs) D67N, K70R, K219QE; and K103N which were slightly more prevalent in DNA than RNA. Subjects who had received cART for at least 5 years were more likely to harbour >2 DRM (p < 0.05) compared to those treated for a shorter period. DRM were more prevalent in this rural setting compared to a neighbouring urban setting.

**Conclusion:**

We found a very high prevalence of NRTI and NNRTI DRM in patients from rural Limpopo settings with different durations of treatment. The prevalence was significantly higher than those reported in urban settings in South Africa. The dominance of NNRTI based mutations late in treatment supports the use of PI based regimens for second line treatment in this setting. The slight dominance of TAMs in DNA from infected PBMCs compared to plasma virus requires further studies that should include cART subjects with suppressed virus. Such studies will improve our understanding of the pattern of drug resistance and dynamics of viral persistence in these rural settings.

## Background

HIV drug resistance remains a major threat to the success of scaling up of combination antiretroviral therapy (cART) in developing countries, especially in Africa where about 60% of HIV-infected individuals were on cART in 2012 [1]. The increased availability of cART has led to remarkable treatment results in most programs in sub-Saharan Africa (SSA), leading to a significant reduction of new infections [2]. Despite this success, some patients with incomplete adherence to treatment protocols might fail therapy and develop resistant viruses [3]. One challenge of cART in SSA has been the inability to routinely monitor virological parameters such as viral load (VL) and drug resistance, which are important to guide patient management and choose the right salvage treatments [4, 5]. Recent WHO recommendations stipulate that VL monitoring be utilized as the preferred method to determine treatment failure in SSA [6]. Within the past years, VL monitoring has in fact been successfully implemented as part of care in South Africa [6].

Sequencing is the most accurate method to track the emergence of DRM and is typically done by Sanger sequencing. However, cost and availability of infrastructure to perform these assays still remain major obstacles [7]. As a consequence, most patients failing therapy remain on first-line therapy for long periods causing an accumulation of mutations, which might lead to the emergence of high-level resistance [8, 9]. This is particularly true for patients who started treatment long before VL was more widely available. Samples from such long term cART patients provide a good source to track previous DRM which can be found only in the cellular compartment.

Genotypic drug resistance studies of subjects with unsuppressed VL have been performed in several SSA countries including South Africa and suggest a high level of DRM as well as some subtype specific mutations [10–16]. For example, previous studies among South Africans reported that infections with HIV-1 subtype C may lead to a rapid emergence of certain non-nucleoside reverse transcriptase (NNRTI) or nucleotide reverse transcriptase inhibitor (NRTI) DRM, which are less common among other subtypes. Specifically, V106M (NNRTI), K65R (NRTI) and N348I are more common among subtype C strains compared to other group M strains [17–19].

The South African treatment protocols are consistent with the 2016 WHO guidelines, which recommend a combination of two NRTIs and one NNRTIs as a first-line regimen and two-NRTI and one protease inhibitor (PI) as second line. According to the April 2014 South African revised guidelines, a preferred first-line regimen could be: tenofovir (TDF)/lamivudine (3TC) + efavirenz (EFV)/nevirapine (NVP), or alternatively abacavir (ABC) + 3TC + EFV; or stavudine (d4T) + 3TC + EFV. For second line treatment, TDF + 3TC + lopinavir/ritonavir (LPV/r); or 3TC + AZT + LPV/r could be used.

Despite the benefits of cART, a significant number of patients still experience treatment failure, associated with the development of DRM due to incomplete adherence to therapy or the presence of transmitted DRM [20, 21]. Given the changing treatment strategies and options, genotypic and phenotypic drug resistance patterns will continue to evolve. Although considerable data on virologic failure in patients with unsuppressed VL on first or second line treatment from South Africa are available, these studies have been mostly done in urban settings [15, 22, 23]. On the contrary, rural settings (making up almost 40% of the South African population) still remain largely understudied. Such rural populations often face significant challenges such as distance to treatment sites and access to virological monitoring. In addition, cultural beliefs often increase stigma and could impact adherence and prevalence of DRM [24, 25]. Two recent studies in Uganda and South Africa reported that the intensity of virological monitoring, frequency and pattern of DRM is influenced by the location (urban or rural) of the treatment site [26, 27]. Such results illustrate that inadequate infrastructure and low per capita income common in rural settings might influence the successful implementation of cART. It is therefore imperative that frequent monitoring of DRM is implemented in order to better understand the relationship between drug resistance development and therapy failures in these rural settings.

The use of integrated proviral DNA rather than RNA isolated from circulating virus to monitor HIV drug resistance in patients failing treatment has been considered as an alternative method for drug resistance genotyping [28]. Sequencing of DNA is also an efficient method to study the archived and persistent viral reservoirs in patients with low or suppressed viral loads [29]. With the current focus on HIV persistence, it is important that studies investigating persisting viral populations during suppression are performed to better understand the viral dynamics and alternative future treatment options.

In this study, we describe the drug resistance profile of HIV-1 infected individuals experiencing unsuppressed VL attending two rural HIV treatment centres; the HIV/AIDS Prevention Group Wellness Clinic (HAPG) in Bela-Bela and the Donald Frazer Hope Clinic (DFHC) in Vhufhuli, both located in the Limpopo Province of northern South Africa. Our findings show a high prevalence of NNRTI and NRTI, but low level PI mutations. DRM were more prevalent in this rural setting than reported for a nearby urban setting in Pretoria. Furthermore, by comparing the DRM (NRTI and NNRTI) in viral RNA as well as proviral DNA among circulating viruses in plasma and peripheral blood compartments in 35 subjects, we found that the DRM in these two compartments were mostly similar with a slight dominance of thymidine analogue mutations (TAMs) in DNA, a result that necessitates further studies with a larger cohort.

## Methods

### Subjects and specimen collection

From July 2013 to December 2015, blood specimens were collected from participating subjects who comprised a random selection of those with unsuppressed viremia at the HIV/AIDS Prevention Group Wellness Clinic (HAPG) in Bela-Bela and the Donald Frazer Hope Clinic (DFHC) in Vhufhuli, in Limpopo Province of rural northern South Africa. All recruited patients met the study criteria of plasma VL > 1000 copies/ml in one of the following ways: (i) two consecutive VL greater than 1000 copies/ml after previous suppression (ii) one VL > 1000 copies/ml after previous suppression followed by a change in treatment (iii) one VL > 1000 copies/ml after 6 months on ART without suppression.

### CD4 count, viral load determination and sample processing

VL and CD4^+^ T cell counts were all performed by the National Health Laboratory Services (NLS) of South Africa. VL were done annually as stipulated by the 2015 South African Treatment Guideline and involved clinic staff usually calling to remind patients of their next VL testing. For drug resistance determination, 5 ml of whole blood was collected from each study participant and transported to the HIV/AIDS and Global Health Research and Training Laboratory at the Department of Microbiology, University of Venda for processing and analyses. On arrival in the laboratory, plasma was separated from cells by centrifuging at 3000 rpm for 5 min and stored at −80 °C in several aliquots. Total cells comprising mostly buffy coats were stored at 4 °C until further processing.

### DNA and RNA extractions

Plasma RNA and proviral DNA from total cells were extracted using Qiagen Viral RNA Mini Kit and Qiagen blood DNA Midi Kit (Qiagen, Valencia, CA, USA), respectively, according to the manufacturers’ protocols. Extracted DNA and RNA from these samples were then used for PCR and RT-PCR, respectively.

### Polymerase chain reaction and sequencing

A fragment of the pol gene approximately 1.5 kb in size (comprising the complete PR and the first 1200 bp of RT) was amplified using a nested PCR protocol described previously [10, 30]. Briefly, cDNA was prepared from viral RNA and used for a nested PCR [30]. Cycling conditions for cDNA and PCRs are available upon request. Amplicons were purified using the Qiaquick PCR purification kit (Qiagen, Valencia, CA, USA) according to the manufacturers’ protocol.

Direct population based Sanger sequencing was performed on an ABI 3730XL platform using the ABI Prism^®^ BigDye™ Terminator v3.1 ready reaction sequencing kit (Applied Biosystems, Warrington, UK). The resulting forward and reverse nucleotide sequence electro-pherograms were assembled, manually edited and translated into predicted amino acids with SeqMan Pro II software, version 8.0 (DNASTAR, Lasergene, USA). Primer sequences used for amplification and sequencing are available upon request.

### Phylogenetic analysis

Phylogenetic analyses were performed to determine the viral subtypes in relation to reference subtypes from the Los Alamos National Laboratory. Sequence alignment of the gene fragments was performed using MUSCLE in MEGA 6 software [31]. The phylogenetic trees were derived using the Maximum Likelihood Model, which calculates the difference in transition and transversion rates. The reliability of the tree was assessed by bootstrapping of 1000 replicates.

### Drug resistance genotyping

For drug resistance genotyping, the newly derived sequences were submitted to the Stanford HIV Drug Resistance Database (http://hivdb.stanford.edu) for examination of drug resistance mutations. Observed predicted DRM were confirmed using the International AIDS Society-USA (IAS) Panel for drug resistance guideline [32]. All sequences associated with high level resistance according to Stanford and IAS algorithms were considered to be resistant.

### Statistical analyses

The Wilcoxon rank sum test was used to compare groups. P-value <0.05 was considered to be significant. Correlation between the rural and urban settings was performed using the Pearson Correlation.

## Results

### Study participants, clinical data and study sites

Subjects for this study comprised patients attending either the HAPG clinic in Bela-Bela or the Donald Fraser Clinic in Vhufhuli, both in the Limpopo Province of northern South Africa. Participation was purely voluntary and included patients who had been on ART for periods ranging from 2 to 10 years. The HAPG in Bela-Bela Clinic currently enrolls almost 1200 patients, while the Donald Fraser Clinic has about 1500 subjects on ART. Sample collection covered a period of 2.5 years; from July 2013 to December 2015. Blood specimens were collected from a total of 72 HIV-1 ART experienced patients who had an unsuppressed viral load >1000 copies/ml (median VL 15,548 copies/ml). The median age of the participants was 37 years (range 5–64), 41 (56.9%) of whom were females (Table 1). The median CD4^+^ cell count was 293 cells/mm^3^. More than 80% of the subjects were classified as CDC (Centers for Disease Control) stage B or C (CD4^+^ T-cell count less than 500 cells/mm^3^). VL and CD4^+^ cell counts were available for all study participants (Table 1). The most common regimens in use were TDF/FTC/EFV (n = 22; 29.3%), TDF/3TC/EFV (n = 13; 18.1%) and TDF/3TC/LPV/r (n = 9; 12.5%; Table 1).

**Table 1:** Demographic, clinical and virological characteristics of 72 patients on ART experiencing an unsuppressed viral load

Parameter	Value
Number of patients	72
Female	41 (56.9)
Male	31 (43.1)
Median age (range) in years	37 (5–64)
Median CD4 count (range) in cells/mm^3^	293 (6–196)
Median plasma viral load (range) in copies/ml	15,548 (50–73,389)
ART regimen at time of genotyping	
TDF/FTC/EFV	22 (29.3)
TDF/3TC/EFV	13 (18.1)
TDF/3TC/LPV/r	9 (12.5)
ABC/3TC/EFV	7 (9.7)
AZT/3TC/LPV/r	5 (6.9)
TDF/3TC/NVP	3 (4.2)
Others^a^	13 (18.1)
Time on ART (months)	
<24	20 (27.8)
25–60	25 (34.7)
>60	27 (37.5)
Clinical stage (CDC) (%)	
A: >500	14 (19.4)
B: 200–500	36 (50)
C: <200	22 (30.6)
Amplified Genotypes (%)	
Subtype C	65 (94.2)
Subtype B	1 (1.4)
Others (K/C, C/B and unclassified)	3 (4.4)

^a^Other regimen combinations included AZT/DDI/EFV, TDF/DDI/3TC, AZT/DDI/LPV/r, AZT/3TC/EFV, ABC/3TC/LPV/r, AZT/3TC/NVP

### HIV genotyping and drug resistance prediction

**Fig. 1:**
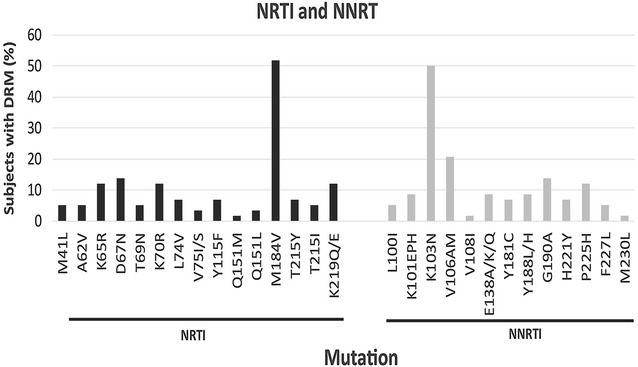
Prevalence and pattern of HIV drug resistance mutations from two treatment centers in rural Limpopo Province. *NRTI* nucleoside reverse transcriptase inhibitor; *NNRTI* non-nucleoside reverse transcriptase inhibitor

PR and RT nucleotide sequences were obtained for 69 plasma samples. The majority of these sequences (n = 65; 94.2%) were subtype C while one each (1.4%) of subtype B, recombinant K/C, recombinant C/B and unclassified were also found (Table 1). Using the Stanford Drug Resistance Database algorithm, 11 (15.9%) sequences did not carry any DRM while 58 (84.1%) had at least one PI, NRTI or NNRTI DRM. Based on drug class, the most common DRM were against NRTI 60.8%; NNRTI 65.2%, and PI 5.8%. The most dominant DRM against the reverse transcriptase inhibitors (RTI), were M184V (51.7%), K103N (50%), V106AM (20.6%), G190A (13.8%), D67N (13.8%), K65R (12.1%) and K219Q/E (12.1%; Fig. 1). The Q151M multi-drug resistant mutation was found in one subject while the transitional mutation (Q151L) which precedes the emergence of Q151M was observed in 2 patients. M230L which causes high level resistance to the entire NNRTI class, occurred in a single case (Fig. 1). E138A/K/Q, a mutation related to the NNRTIs etravirine (ETR) and rilpivirine (RPV) was found in 8.6% of subjects, although these drugs are not currently available and not part of the standard treatment regimen in South Africa. Excluding E138A/K/Q among NNRTI mutations reduced the NNRTI resistance prevalence from 65.2 to 62.5%. Among EFV exposed patients, a relatively high prevalence of P225H (12.1%), was found in combination with K103N, confirming previous observations that this mutation rarely occurs independently. Four cases of the H221Y (7%; Fig. 1) DRM were present, always in combination with Y181C. Although H221Y has minimal detectable effect on NNRTI susceptibility, it has been found to frequently occur in combination with Y181C in RPV exposed patients [33]. However, in the present study H221Y was identified only in patients with EFV exposure.

Three subjects carried the accessory mutation A62V that has been reported to be widespread among subtype A viruses from countries of the former Soviet Union [34]. A62V often occurs in combination with multi-resistance NRTI mutations K65R and Q151M. In this study, all the A62V mutations described were linked to K65R, but not to Q151M [35]. Out of the five patients who carried multiple thymidine analogue mutations (TAMs) only one had the K65R mutation, suggesting that these TAMs likely resulted from previous AZT or d4T treatment and not exposure to TDF. TAMs were dominated by D67N (13.8%), followed by K219Q/E (12.1%) and K70R (12.1%) with a low occurrence of T215Y/F (6.9%). The T215Y/F mutation seem to be subtype specific as it was recently found as the dominant TAM in 33.6% of non-subtype C infected study subjects in five Central African countries [36]. Interestingly, among the viruses with mutations at this position, tyrosine (Y) was the dominant amino acid (6.9%; T215Y) compared to the complete absence of phenylalanine (F) (0%; T215F; Fig. 1). Five percent of viruses carried an isoleucine (I, encoded by ATT, ATC) mutation at position 215 (215I) which resulted from a single nucleotide changing threonine (ACT, ACC) to isoleucine. The 215I mutation seems to be transitional (intermediate) to T215F (phenylalanine-TTT, TTC).

**Fig. 2:**
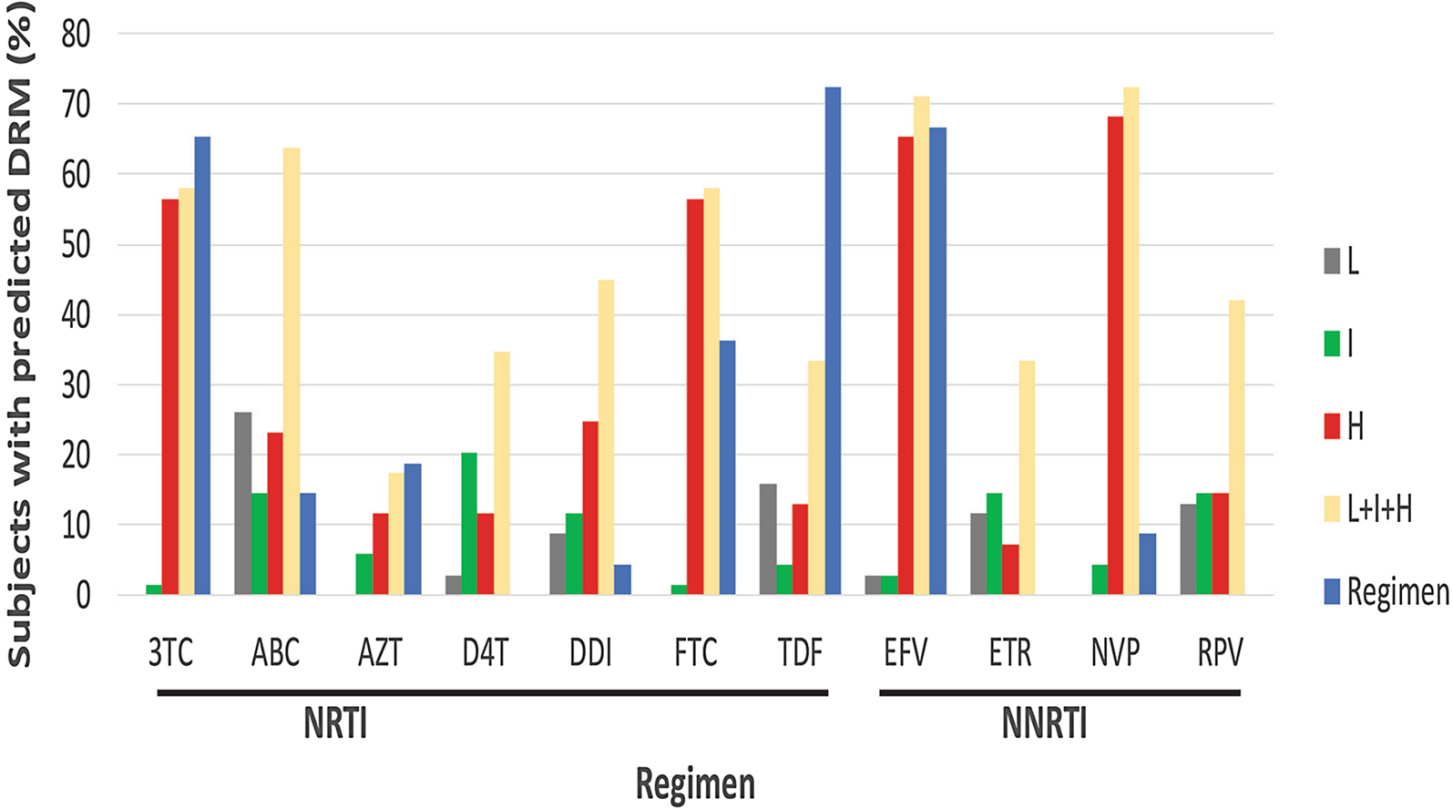
Prevalence of genotypic drug resistance to various antiretroviral drugs. NRTI, nucleoside reverse transcriptase inhibitor; *NNRTI* non-nucleoside reverse transcriptase inhibitor, *3TC* Lamivudine, *ABC* Abacavir, *AZT* Zidovudine, *d4T* Stavudine, *ddI* didanosine, *FTC*, Emtricitabine, *TDF* Tenofovir, *EFV* Efavirenz, *ETR* Etravirine, *NVP* Nevirapine, *RPV* Rilpivirine, *L* Low, *I* Intermediate, *H* High level resistance

Considering that the history of ART influences circulating DRM, we grouped these DRM using the Stanford Drug Resistance algorithm into high, intermediate and low level resistance mutations and compared them to the antiretroviral drugs that these patients were receiving at the time of testing (Fig. 2). The prevalence of the RTIs administered to patients at the time of this study were in the following order: TDF > EFV > 3TC > FTC > AZT > ABC > NVP > ddI (Fig. 2). Lopinavir boosted ritonavir (LPV/r) was the most commonly used PI 20/69 (29%) of study participants (Table 1). However, when we compared the prevalence of the predicted high and medium level DRM to the drugs in use, the highest level of resistance was to NNRTIs, (NVP and EFV) in accordance with their low barrier to resistance. The rank order of the predicted frequency of resistance to the cART in use were as follows: NVP > EFV > ABC > 3TC > FTC > ddI > d4T > TDF > AZT. It is interesting to note that although TDF was the most frequently used drug, it had one of the lowest number of DRM frequencies (high resistance barrier; Fig. 2). Abacavir (ABC) is a recommended component of the first line therapy provided to children and adolescents who made up 25% of the study participants. A relatively high level (>60%) of ABC DRM was observed among study subjects, clearly higher than the number of subjects on ABC. Some of the ABC resistance could also be attributed to its cross resistance to ddI, which shares a similar DRM pattern. Predicted resistance was highest among patients whose regimen included NVP and EFV, two NNRTIs which are also components of first line treatment. Two (3.0%) subjects harbored major PI DRM (D30N, M46I and V32I). T74S, a minor PR mutation, was detected in 45 (65.2%) of the 69 participants. LPV/r was administered as part of the first line treatment to children and adolescents or as second line to adults.

**Fig. 3:**
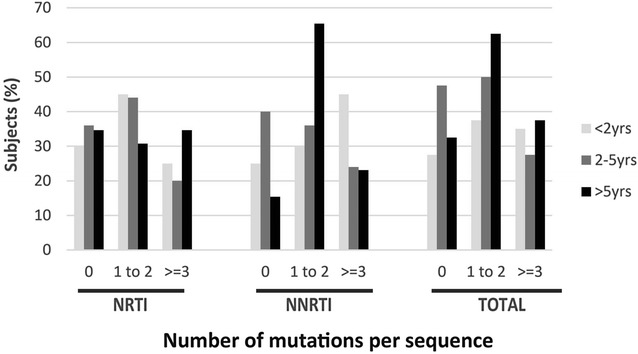
Comparing the frequency of NRTI versus NNRTI drug resistance mutations over time. *NRTI* nucleoside reverse transcriptase inhibitor, *NNRTI* non-nucleoside reverse transcriptase inhibitor

More than two-thirds of subjects had been on ART for at least 24 months, the majority (62%) of these had been exposed to more than one drug regimen. We therefore assessed the frequency of current NRTI and NNRTI DRM in correlation with the length of time of cART exposure (Fig. 3). Subjects were categorized based on the number of DRM (0, 1–2, and ≤3) and the length of time on cART (<2, 2–5 and >5 years; Fig. 3). The distribution of NRTI DRM was mostly similar over time (<5 years) for subjects harbouring less than 3 mutations (Fig. 3). After 5 years of treatment, the number of subjects carrying >3 NRTI mutations were higher (but not significantly) than those with <2 mutations suggesting an accumulation of DRM overtime. For the NNRTIs, one or two DRM (either K103N, V106M or G190A) were the most common and were more prevalent in subjects that had been on treatment for >5 years (p < 0.05; Fig. 3).

Healthcare facilities and living standards differ greatly between urban and rural settings in SSA countries including South Africa. Recently, a higher level of DRM was observed in a rural area of KwaZulu-Natal compared to an urban cohort in Pretoria [26]. As the rural settings of Limpopo and KwaZulu-Natal differ somewhat especially in population density, we compared the DRM data from the Pretoria study to those from our study (Table 2). Twenty NRTI and NNRTI DRM positions were considered and the frequency of these mutations in rural versus urban settings were compared. More than half of these mutations (K65R, D67N, K70R, L74V, V75I/S, Y115F, K219Q/E, K101E/P/H, K103N, V106AM, Y181C and Y188L/H) were significantly more prevalent (p < 0.05) in the rural settings of Limpopo than in urban Pretoria (Table 2). The prevalence of these DRM in the two rural sites of Limpopo and KwaZulu-Natal correlated well (r^2^ = 0.93). Exceptionally, the M184V mutation prevalence in rural Limpopo was more closely related to urban Pretoria (51.7% vs. 53.4%) than rural Kwazulu-Natal (79.8%; Table 2).

**Table 2:** Comparison of the NRTI and NNRTI DRM prevalence from Limpopo (rural), KwaZulu-Natal (rural) and urban (Pretoria)

Mutation	Limpopo	Kwazulu-Natal (rural)^a^	Pretoria (urban)^a^	p-value (Limpopo vs. Pretoria)^b^
M41L	5.2	7.1	5.8	1
A62V	5.2	2.6	1	0.89
K65R	12.1	13.1	1.9	<*0.01*
D67N	13.8	16.8	5.8	*0.014*
K70R	12.1	14.3	7.8	*0.039*
L74V	6.9	2.6	1	*0.023*
V75I/S	3.4	3.8	0	0.089
Y115F	6.9	5.3	0	*0.014*
Q151M	1.7	1.8	1.9	0.91
M184V	51.7	79.8	51.7	1
T215Y	6.9	12.5	8.7	0.5
K219Q/E	12.1	13.3	5.8	<*0.01*
L100I	5.2	3	2.9	0.09
K101EPH	8.6	1.8	1	<*0.01*
K103N	50.0	44.5	34	<*0.01*
V106AM	20.7	28.9	34	<*0.01*
V108I	1.7	10.5	3.9	0.17
Y181C	6.9	12.5	11.7	*0.03*
Y188L/H	8.6	10.1	2.9	*0.01*
G190A	13.8	15.8	9.7	0.05

^a^Data obtained from study by Rossouw et al. [26]

^b^The p-values represent significance (in itatlics) of comparisons between the rural sites from this study (Limpopo) and the urban site (Pretoria) from the study by Rossouw et al. [26]

**Fig. 4:**
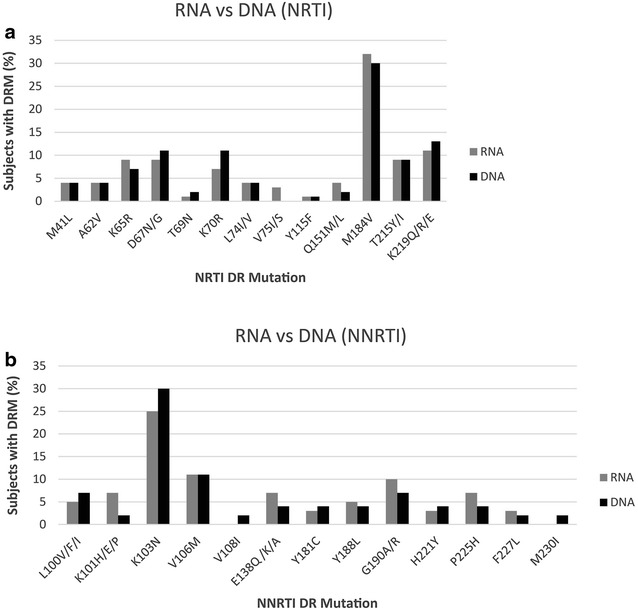
Comparing the DRM in RNA (plasma) and DNA (peripheral blood mononuclear cells). **a** NRTI. **b** NNRTI

In order to assess if integrated proviral DNA can be used to analyze drug resistance in patients failing therapy, we amplified and sequenced the PR and RT genes of 35 subjects from proviral DNA (PBMC). DNA sequences generated were compared to corresponding paired RNA sequences (Fig. 4a, b). Of the 13 different NRTI DRM that were detected by any method in this study, nine DRM were found in the same or greater proportion of the population using the viral RNA assay as compared with the proviral DNA assay (Fig. 4a, b). Interestingly, four DRM were detected in a greater proportion of the population using the DNA assay, of which three were TAMs; namely D67N, K70R and K219Q/R/E (Fig. 4a). A similar observation was made among the NNRTI mutations in which 8/13 of the DRM were found to be either identical to or more prevalent using the viral RNA assay than the proviral DNA assay. The exception was the K103N mutation which was significantly more prevalent (p < 0.05) in assays of proviral DNA compared to assays using viral RNA (Fig. 4b).

## Discussion

This study was performed among patients failing treatment in two treatment centres in rural South Africa: the HAPG Wellness Clinic which started cART administration in 2000, 4 years before the roll-out of antiretroviral therapy in the public health sector in South Africa; and the Donald Fraser Clinic which started providing treatment in 2004. Although both sites have been implementing VL and CD4^+^ T-cell count monitoring, according to national guidelines, and as part of HIV management for several years, the prevalence of DRM was still high. The M184V mutation was the most prevalent DRM, observed in 52% of study subjects. This coincided with the extensive use of 3TC, surpassed only by the use of TDF and EFV in the population. Interestingly, TDF mutations were one of the least common DRM in the study despite its frequent usage, supporting its inclusion as the main backbone during first line treatment.

Although currently used cART is effective for all group M subtypes, the pattern of drug resistance differs between subtypes [10, 36]. Our study confirmed the dominance of K65R and V106M, two mutations which are most common among subtype C strains [36, 37]. In fact, very recently in a cohort of more than 3700 subjects failing cART in several West and Central Africa countries, these two DRM were found to be more prevalent among subtypes C strains compared to CRF02_AG, CRF06cpx, and subtypes A and G [17, 18, 36]. This dominance has been attributed to the genomic sequence of subtype C, which requires only a single nucleotide change at these codon positions to transition to a drug resistant amino acid residue [37]. Similarly, we speculate that the occurrence of the E138A/K/Q mutation could be due to the codon usage of subtype C RT at this position which is GAA versus GAG in other group M strains. In fact, in the Stanford Drug Resistance database, subtype C strains from drug exposed patients have a higher prevalence of the E138A/K/Q mutation compared to other group M strains (8–9.5% vs. 2–5%). Further verification of such subtype-specific differences in the pattern of resistance mutations would clearly be of great significance for treatment management and also for a better understanding of the overall resistance mechanism.

The high occurrence of both K103N and V106M could be a reflection of the wide use of EFV and NVP as part of the first line therapy. In addition, the high levels of V106M mutations could also be attributed to subtype-specificity, since this mutation had been found most frequently among subtype C [17, 36]. It seems quite clear that NNRTIs should not be used for second line treatment in rural South African populations. This conclusion is supported by the fact that a significant number of the patients we studied already show resistance to newer NNRTIs (ETR and RPV), which have not even been introduced in South Africa.

The selection of TAMs such as D67N, M41L and T215Y could be attributed to treatment with the thymidine analogues AZT and d4T, which also cause cross resistance to other NRTIs [8]. Similarly, patients harbouring TAMs also had high level resistance to NNRTIs (EFV and NVP) and 3TC supporting the point that they were likely failing thymidine containing ART at the point when TDF replaced AZT in the national guidelines [38]. TAMs have been reported to be more common among CRF06cpx strains than other HIV-1 subtypes or CRFs [10, 36]. In this study, there was a noticeable absence among subjects of the L210W and very low occurrence of the M41L DRM, both components of the Type 1 TAMs pathway (M41L, L210W and T215Y). Although, this could likely be related to the reduced usage of d4T, recent reports suggest that compared to other dominant HIV-1 genotypes (subtypes A, CRF02_AG and CRF06cpx), subtype C show the lowest prevalence of M41L DRM [36]. The absence of M41L and L210W was initially reported from a subtype C cohort in Botswana where the most common TAM pathway was D67N, K70R and T215Y [39]. In our study, we observed that the four TAMs (D67N, K70R, T215Y and K219Q/E) which make up the Type 2 TAM pathway, occurred more frequently and correlated well with the usage of AZT. Although we could not state categorically the order of emergence of these mutations as this was a cross sectional study, the mutations D67N and K70R were the most frequent single occurring TAMs. Both AZT and d4T have been reported to cause TAMs at an equal rate [40].

The majority of participants in this study were adults >18 years old. This is not surprising given that treatment of children and adolescents with cART has not been vigorously pursued in SSA until recently. Out of the 13 subjects <18 years old with unsuppressed virus, DRM could be detected in 70% (6 were TAMs and 3 were either NNRTI or other NRTI). DRM has been reported to be highly prevalent among African children with unsuppressed VL [41]. In Uganda and South Africa, DRMs were higher in children from rural compared to urban clinics indicating a better management and/or easier adherence in urban clinics [26, 27]. Due to the low number of children and adolescents in this study, we could not independently confirm this. With the increasing usage of cART in children and adolescents in SSA, more longitudinal studies are required to better understand the pattern of DRM among this group.

The observed high levels of DRM in these rural settings could be linked to several factors. First, the availability of transportation to treatment centers is generally significantly reduced in rural areas compared to urban settings. Secondly, shortage of cART supplies are common occurrences in rural areas. Finally, cultural differences or stigma might affect the open administration of cART especially among women, a phenomenon that is more strongly present in rural areas. Another factor that likely influenced the high rates of DRM in one of the study sites (HAPG) was the inadequate planning and poor follow-up by health authorities. In 2009, at the HAPG privately run facility in Bela-Bela, patients’ healthcare providers were switched as patients were transferred from HAPG to a newly opened nearby Public Health Clinic (PHC). Records dating from the beginning of cART therapy at HAPG in 2004 showed good patient follow-up and less treatment failure. However, after the massive patient transfer in 2009 to the PHC and their return to the HAPG private clinic in 2012, the rate of virologic failure increased significantly among returning patients. Despite the high prevalence of DRM observed in this study, the number of patients with detectable VL, but no DRM was similar to previous reports (16% vs. 19–36% respectively) in some urban centres in South Africa [22, 36, 42, 43].

There are few reports on the prevalence and pattern of DRM in rural South Africa [26, 30, 44]. A recent study compared the frequency of DRM from rural KwaZulu-Natal and urban Pretoria, and found a clear difference between these two sites [26]. In our current study, we confirmed the findings of Rossouw et al. Notably, the number of DRMs at the two sites in rural Limpopo were generally higher than in neighboring urban Pretoria (Table 2). Only two DRMs were substantially more common in Pretoria as compared with the Limpopo population. The mutations V106A/M and Y181C were 13.3 and 4.8% more common in Pretoria than in Limpopo, respectively. Both mutations are associated with exposure to NVP [45]. At the time of sequencing, the Pretoria subjects were much more likely to be exposed to NVP than were the subjects in Limpopo (32.4% vs. 5%). Although subjects in these rural settings would be predicted to respond to the ART regimens that are currently in use, we suggest that the WHO recommendation that includes a PI in the second line treatment and close monitoring be strictly observed in order to reduce the transmission of DRM. This recommendation is supported by the very low level of PI DRMs observed in this study.

We compared DRM from both plasma (RNA) and cellular (proviral DNA) compartments from patients experiencing unsuppressed viral load who had received treatment for varying periods, ranging from 1 to 10 years. Plasma is routinely used for drug resistance testing, because viral RNA reflects the current replicating virus in blood. The prevalence of DRM in RNA and DNA was mostly similar with the exception of the TAMs, which were more prevalent in DNA than RNA. The persistence of some TAMs in DNA could be explained by the formerly common administration of NRTI (AZT and d4T) regimens. Of note is the fact that d4T is gradually being phased out and was not included in any of the current treatment regimens received by the study subjects. Three viral RNA and two proviral DNA derived sequences from patients who had never been treated with ddI, ABC, or TDF harboured a K65R mutation.

It is important to point out that our analyses were done using routine population-based Sanger sequencing that does not allow the detection of minority viral variants which comprise less than 20% of the viral population in a patient sample [46, 47]. Recent methods such as next generation sequencing would most likely detect minor drug resistant variants at levels as low as 1% [47, 48]. Considering the fact that undetected drug resistant minority variants may persist when cART is discontinued or changed, it will be worthwhile to perform comparative RNA/DNA drug resistance studies using more sensitive next generation sequencing [47]. Although advances in cART has improved the lives of HIV infected patients, current regimens have failed to eliminate latent reservoirs, and it is acknowledged that eradication of the virus is not possible with current cART regimens alone [49]. Furthermore, the efficacy of switching drugs for patients experiencing a rebound in VL will depend mainly on the number of DRM persisting in the proviral reservoir, following previous therapeutic failures [50, 51].

Overall, these results clearly show that patients receiving cART, but experiencing therapeutic failure, harbour diverse drug-resistant variants, which in some cases persisted in the cellular blood compartment. Detection of this persisting variants can be improved by using more sensitive methods such as next generation sequencing. The proviral variants observed in this study closely resemble the population found in the plasma compartment (viral RNA) with the exception of some persistent TAMs; confirming the known fact that proviral DNA constitutes a reservoir for drug-resistant variants which might replenish plasma pool during suboptimal therapy.

Two limitations should be noted in our investigation: first, this was a cross-sectional study in which data on adherence to treatment was not available; as a result, it was not possible to associate the observed virologic failure to poor adherence. Second, information on prior exposure to NVP in the early days of mother-to-children transmission (MTCT) prevention efforts was also not available. The use of nevirapine monotherapy could have potentially impacted on the observed level of NNRTI mutations particularly K103N.

In conclusion, this study suggests that there is a high level of DRM in rural South Africa and confirms recent observations from KwaZulu-Natal that DRMs are more prevalent in rural than urban areas. It also confirms that long term ART with no virological monitoring could lead to an accumulation of DRMs especially of the NRTI class. Although plasma and PBMCs presented largely identical DRMs, the later compartment may act as an archive of drug resistant variants, especially TAMs, an observation that needs further studies with a larger sample size including subjects with suppressed virus. Such studies could shed more light on the persistence of DRMs and their impact on HIV treatment and cure.
